# Methionine restriction in cancer: a dietary insight for therapy

**DOI:** 10.3389/fnut.2026.1730639

**Published:** 2026-02-03

**Authors:** Xing Tian, Gengjun Zhu, Yuhua Zhang, Ning Liu

**Affiliations:** 1 Central Laboratory, The Second Hospital of Jilin University, Jilin University, Changchun, China; 2 Key Laboratory of Zoonosis Research, Ministry of Education, Jilin University, Changchun, China

**Keywords:** combination therapy, epigenetics, MAT2A, methionine restriction, MTAP

## Abstract

**Background:**

Methionine, one of the essential amino acids that needs to be obtained through protein-rich diet, provides important sulfur elements for the human body, which is crucial for protein synthesis, antioxidant and metabolic regulation. Many tumors develop a metabolic dependency due to the lack of a working methionine salvage pathway, which can be targeted by methionine restriction (MR).

**Results:**

The core mechanism of MR lies in disrupting one-carbon metabolism and epigenetic regulation that rely on methionine, depleting the crucial metabolite S-adenosylmethionine (SAM), thereby inhibiting histone/DNA methylation, disrupting redox homeostasis, and ultimately inducing cell cycle arrest and apoptosis. Substantial evidence indicates that MR, achieved by specific metabolic enzyme inhibitors or diet with special formulations, can intervene in tumor progression from a metabolic standpoint. Additionally, combining methionine restriction with existing treatments can achieve satisfactory outcomes in the clinical management of various tumors.

**Conclusion:**

This article provides insights into the research and its translational potential of methionine restriction as a promising strategy for cancer treatment, with the emphasis on the contributions that advance the field and better serve the clinical research community.

## Graphical Abstract

**Figure:**
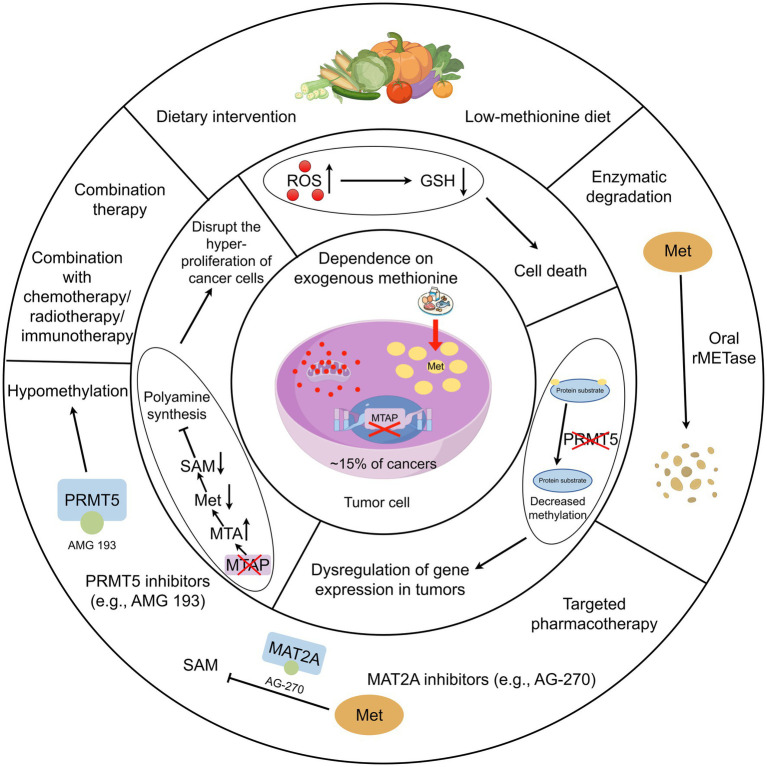
Targeting the methionine addiction of cancer: a precision strategy against a metabolic vulnerability. Reactive oxygen species (ROS), Glutathione (GSH); S-adenosylmethionine (SAM), S-adenosylhomocysteine (SAH), Protein arginine methyltransferase 5 (PRMT5), Methylthioadenosine phosphorylase (MTAP), Methylthioadenosine (MTA), Methionine (Met), Oral recombinant methioninase (Oral rMETase), Methionine adenosyltransferase 2A (MAT2A), MAT2A inhibitor AG-270 (AG-270), PRMT5 inhibitor AMG 193 (AMG 193).

## 1 Introduction

A century ago, Sugimura et al. discovered that restricting the intake of specific amino acids (particularly methionine, Met) could significantly inhibit tumor growth in rats (1). This breakthrough study revealed a distinct dependence of cancer cells on exogenous methionine. In contrast, normal cells can synthesize methionine from homocysteine using methionine synthase, with cobalamin and 5-methyltetrahydrofolate serving as essential cofactors (2).

Studies in 1976 further demonstrated that although intracellular Met levels in cancer cells are normal or even elevated, their proliferation remains absolutely dependent on exogenous Met, a contradictory occurrence known as the ‘Hoffman effect’ (3–5). Many tumors develop a metabolic dependency due to the lack of a working methionine salvage pathway. Approximately 15% of cancers, including glioma and pancreatic cancer, show a loss of Methylthioadenosine Phosphorylase (MTAP) (5). This loss primarily occurs due to co-deletion of the Cyclin-Dependent Kinase Inhibitor 2A (CDKN2A) gene—which encodes the p16 tumor suppressor protein—or through promoter methylation of the MTAP gene (5). MTAP deficiency prevents tumors from recycling the polyamine metabolism byproduct methylthioadenosine (MTA) back into Met, forcing reliance on exogenous Met, while simultaneously increasing sensitivity to purine synthesis inhibitors (6–8). In cancer cells, Met deprivation induces S/G2 phase cell cycle arrest (9, 10), depletes glutathione triggering apoptosis (11), and inhibits O6-methylguanine-DNA methyltransferase (MGMT) impairing DNA repair (12); these pathways enhance the efficacy of chemotherapy/radiotherapy and improve treatment outcomes *in vivo* (13, 14). Met in tumor biology can both support rapid proliferation as an amino acid for protein synthesis and regulate epigenetic modifications (DNA/RNA/protein methylation) and redox homeostasis as a precursor to S-adenosylmethionine (SAM). Normal cells convert only about 10% of Met to SAM, whereas tumors preferentially utilize SAM-driven methylation programs to maintain their malignant phenotype (5, 15).

Intervention strategies targeting tumor metabolism can be traced back to Otto Warburg’s theory of aerobic glycolysis in cancer (the “Warburg effect”) proposed in the 1920s (16). Over the past 50 years, consistent preclinical evidence has shown that methionine restriction therapy (MRT) exerts broad-spectrum antitumor activity against both solid tumors (e.g., colorectal and melanoma) and hematological malignancies (4, 17, 18). MRT works primarily by regulating SAM-dependent methylation modifications and cellular redox homeostasis (4, 18). Clinical translational research demonstrates that medical-grade low-methionine diets (Met < 0.1% w/w) substantially decrease serum methionine levels in patients (Δ[Met] > 60%) and improve the effectiveness of platinum-based chemotherapy by causing tumor cells to halt their cycle and preventing the repair of DNA damage (19, 20). This review systematically analyzes the dual role of methionine-restricted diets in tumor development: on one hand, acting as a metabolic vulnerability target to induce tumor-specific death, and on the other hand, remodeling the tumor microenvironment through epigenetic reprogramming, while focusing on the challenges and optimization strategies in its clinical translation.

## 2 Methionine metabolism

**Figure 1:**
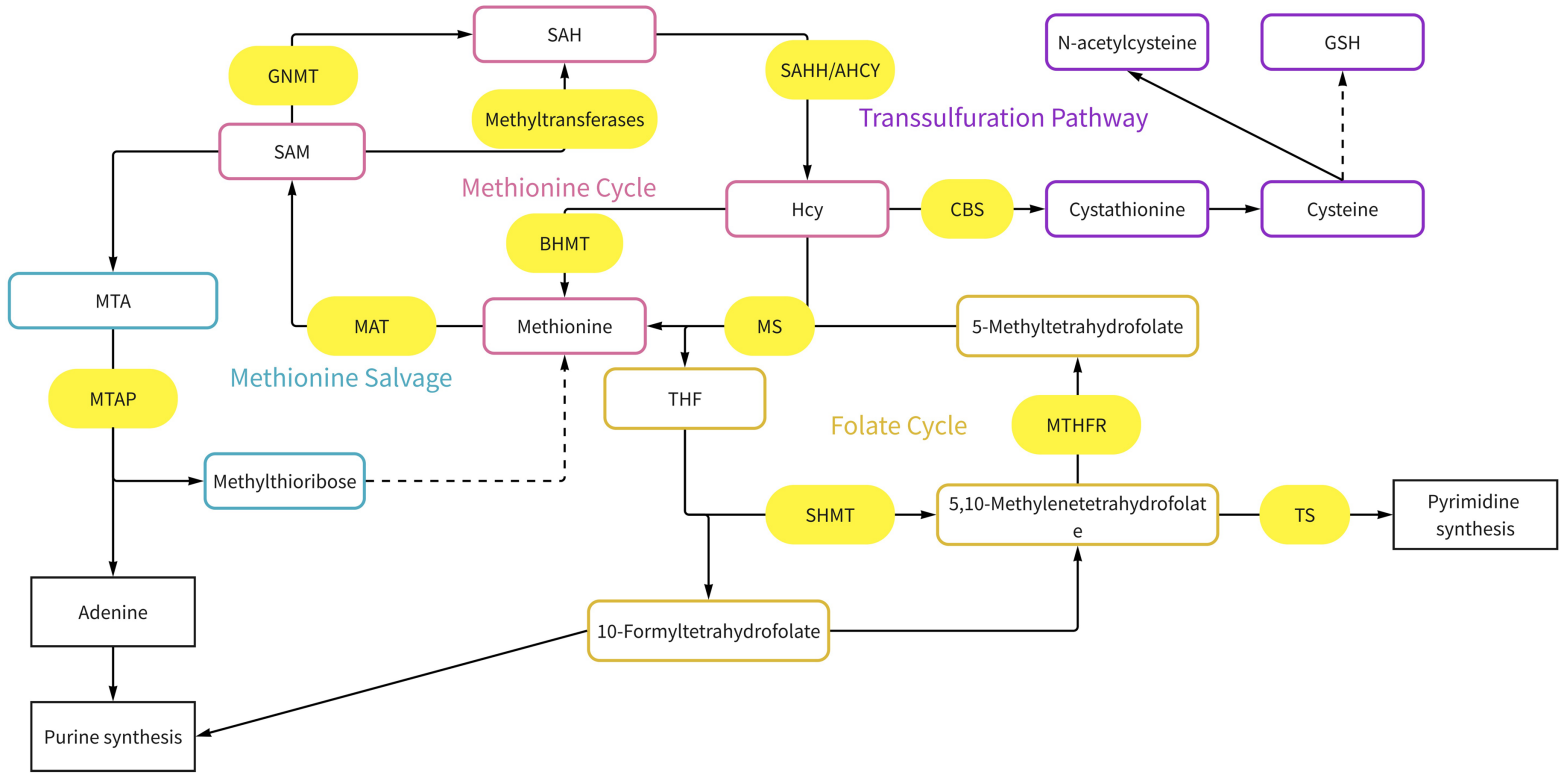
Methionine metabolism. Methionine metabolism is interconnected with multiple metabolic pathways, including the folate cycle, transmethylation, and transsulfuration pathways. Methionine adenosyltransferase (MAT), *S*-adenosylmethionine (SAM), *S*-adenosylhomocysteine (SAH), adenosylhomocysteinase(AHCY), methionine synthase (MS), betaine-homocysteine methyltransferase (BHMT), methylthioadenosine phosphorylase (MTAP), cystathionine *β*-synthase (CBS), glycine *N*-methyltransferase (GNMT), dihydrofolate reductase (DHFR), serine hydroxymethyltransferase (SHMT), methylenetetrahydrofolate reductase (MTHFR), tetrahydrofolate (THF), methylthioadenosine (MTA).

In mammals, methionine is a crucial amino acid. The methionine cycle, as a key pathway of its metabolism, together with the folate cycle, constitutes one-carbon metabolism, regulating nucleotide biosynthesis (21, 22) (Figure 1). In the methionine cycle, methionine is first converted into SAM by the enzyme methionine adenosyltransferase (MAT). SAM, which contains an activated methyl group, is the body’s main direct methyl donor. Its activated methyl group is primarily used to modify histones and DNA. Methyltransferases aid the methylation process by using SAM as a direct source of methyl groups. The methyl group transfer to the target molecule transforms SAM into S-adenosylhomocysteine (SAH), which subsequently undergoes a deadenylation reaction to yield homocysteine (23). Concurrently, the salvage pathway enables the regeneration of methionine from SAM through the intermediate methylthioadenosine, under the sequential catalysis of MTAP and other enzymes. In approximately 15% of cancer cases, the MTAP gene is co-deleted due to its proximal location to the CDKN2A gene. This genomic alteration enhances the sensitivity of cancer cells to low concentrations of SAM, a phenomenon observed in nasopharyngeal carcinoma (22, 24, 25). However, not all cancer cells respond to low SAM concentrations. In melanoma, the MeWo cell line is not sensitive to low SAM and can be rescued by homocysteine (26). Homocysteine metabolism involves two pathways: remethylation, where homocysteine is converted back to methionine using a methyl group from N5-CH3-TH4, completing the methionine cycle; and trans-sulfuration, where cystathionin-*β*-synthase (CBS) catalyzes homocysteine to produce glutathione (22, 27). The trans-sulfuration pathway primarily serves to buffer oxidative stress in cancer cells, while glutathione depletion may be a contributing factor to oxidative stress.

In the folate cycle, tetrahydrofolate initially transforms into 10-formyltetrahydrofolate (10-formyl-THF). 10-formyl-THF accepts a methyl group from serine to form 5,10-methylenetetrahydrofolate (5,10-MTHF) (27). The latter can be converted to 5-methyltetrahydrofolate, which provides a methyl group for the regeneration of methionine, and regenerates tetrahydrofolate (28). The folate and methionine cycles are interconnected primarily through methionine synthase (MS). Through the catalytic activity of methionine synthase, a methyl group is transferred from methyltetrahydrofolate to homocysteine, leading to the synthesis of methionine and the conversion of methyltetrahydrofolate to tetrahydrofolate (FH4). In the liver and other tissues, betaine can act as a methyl donor to convert homocysteine into methionine (29).

The distinct reliance of cancer cells on exogenous methionine constitutes a primary metabolic characteristic. Consequently, restricting methionine intake may exert diverse effects on their metabolic network. To buffer the oxidative stress generated by rapid growth, division, and metabolic abnormalities, cancer cells have a higher demand for glutathione than normal cells, which leads homocysteine to favor the trans-sulfuration pathway over methionine regeneration (30). Furthermore, when methionine is deprived, the endogenous methionine in cancer cells is preferentially used for protein synthesis. Both of these factors reduce the flux through the methionine cycle. SAM, also known as active methionine, is generated when the terminal methyl group of methionine becomes an activated methyl group under the action of MAT, and it is an important methyl donor in the body. Under MR, glutathione levels are temporarily depleted, contributing to the accumulation of oxidative stress. In lung tumors, oxidative stress elevates cystathionine-*β*-synthase (CBS) activity while reducing MAT activity, worsening methionine and SAM deficiency (31). Under methionine deprivation, SAM levels consequently decrease. The reduction of this crucial intracellular methyl donor leads to global hypomethylation. To sustain proliferation, cancer cells prioritize methyl group recycling from existing histone marks, thereby reducing certain histone methylation marks, such as H3K27me3, and inhibiting cancer development.

## 3 Antitumor mechanisms of methionine restriction

### 3.1 Methionine restriction exerts antitumor effects by influencing epigenetic regulation

In the field of cancer epigenetics, DNA methylation exhibits dual dysregulation characterized by genome-wide hypomethylation and specific hypermethylation of approximately 60–70% of gene promoters containing Cytosine-phosphate-Guanine (CpG) islands, particularly those related to tumor suppressor genes, leading to their transcriptional silencing (32–34). This aberrant methylation pattern, in conjunction with histone modifications such as H3K36me3, forms the essential epigenetic foundation required to sustain the proliferative state of cancer cells (32, 33). The establishment and maintenance of such an epigenetic landscape require extensive DNA, RNA, and histone methylation, a process that consumes massive amounts of S-adenosylmethionine (SAM). Rapid proliferation requires the massive synthesis of new proteins, nucleic acids (RNA and DNA), and phospholipids, and the synthesis and modification processes of these substances also require the support of methylation reactions (35). SAM, as the most important methyl donor within the cell, regulates gene expression by providing methyl groups via MAT to catalyze relevant intracellular reactions, which is crucial for driving the development of various cancers (36–40).

MR in gastric cancer cells results in decreased expression of the long non-coding RNA plasmacytoma variant translocation 1 (lncRNA PVT1) (41, 42). Lin Xin et al., using methionine-deficient culture in nude mouse gastric cancer models and human gastric cancer xenografts, found that long non-coding RNA PVT1 levels were downregulated, subsequently affecting BCL2/Adenovirus E1B 19 kDa Interacting Protein 3 (BNIP3) expression (41). Sarah Séité et al. demonstrated that gastric cancer cell lines MKN45 and SGC-7901 cultured in methionine-deficient medium exhibited significantly reduced cell viability and proliferative capacity (42). The study further revealed that under methionine deprivation, the long non-coding RNA PVT1 could interact with DNA Methyltransferase 3 (DNMT3) to modulate the DNA methylation level at the promoter region of the pro-apoptotic gene BNIP3. This finding indicates that methionine can participate in the regulation of gastric cancer progression by orchestrating specific DNA methylation modifications through “lncRNA-protein” interactions, specifically via the PVT1-DNMT3 axis. Furthermore, another study reported that combining methionine deprivation with a histone deacetylase 2 (HDAC2) inhibitor effectively reduced the level of the repressive histone mark H3K27me3 while increasing the level of the activating mark H3K27ac at the promoter of the epithelial marker E-cadherin. This epigenetic remodeling can reverse the epithelial-mesenchymal transition process, thereby significantly inhibiting the migration, invasion, and metastatic capabilities of gastric cancer cells (43).

Furthermore, MET directly regulates the methylation modifications of DNA and histones via metabolic processes. In the context of lung cancer, this dysregulation is marked by pronounced abnormalities in DNA methylation, typically characterized by a coexistence of genome-wide hypomethylation alongside specific hypermethylation of tumor suppressor gene promoters, including Death-associated protein kinase (DAPK), Ras association domain family member 1A (RASSF1A), and Retinoic acid receptor *β* (RARβ), among others (44, 45). This precisely reflects the special gene expression pattern of cancer cells. Consequently, lung cancer cells can evade apoptosis and proliferate malignantly. MR can reduce H3K4me3, H3K9me2, H3K27me3, and H3K36me3 methylation, enhancing the drug sensitivity of lung cancer cells (46). Disrupting the promoter hypermethylation status of tumor suppressor genes impairs the malignant proliferation of cancer cells, dealing a fatal blow to cancer development. The study by Ting Li et al. demonstrated that a methionine-restricted diet inhibits tumor growth in multiple mouse models and enhances both the number and cytotoxic function of tumor-infiltrating CD8^+^ T cells (as indicated by upregulated expression of Granzyme B and Interferon-gamma) (47). The research further elucidated the underlying molecular mechanism: methionine restriction or genetic knockout of YTH Domain Family Protein 1 (YTHDF1) can synergistically block tumor immune escape at the metabolic level (by reducing the supply of SAM) and the translational level (by abolishing (Pandit, 2023 #179)m^6^A-dependent translational enhancement), respectively. These interventions effectively suppress the efficient synthesis of immunosuppressive proteins such as programmed death-ligand 1 (PD-L1) in tumor cells. As the tumor immunosuppressive barrier is weakened, CD8^+^ T-cell function is restored, allowing the cells to re-infiltrate the tumor microenvironment. Moreover, the study confirmed that combining this metabolic intervention with a PD-1 inhibitor generates a powerful synergistic anti-tumor effect, leading to more effective suppression of tumor growth. In mixed lineage leukemia, methionine adenosyltransferase 2A (MAT2A) is significantly overexpressed (48, 49). The MAT2A inhibitor PF-9366, employed to simulate MR, was tested on CRISPR/Cas9-engineered Mixed-Lineage Leukemia (MLL)-AF4and MLL-AF9 fusion gene models, MLL-rearranged (MLLr) cell lines (SEM t(4;11) and THP-1 t(9;11)), and the non-MLLr cell line SKM-1, using healthy cells as controls (48). The results indicated that when MAT2A is inhibited, SAM lacks a replenishment source. This can reduce MLLr cell proliferation and viability, and decrease SAM levels and histone methylation (50). Decreased histone methylations, including H3K4me3, H3K79me1, H3K79me2, and H4R3me2, can suppress cancer cell malignancy. Because the histone H3K27 methyltransferase EZH2 has a higher affinity for SAM (with a lower Km value) than the H3K4 methyltransferase MLL1, under conditions of limited SAM availability, EZH2 can utilize the substrate more efficiently and maintain its activity relatively well, whereas MLL1 activity is more severely suppressed (51). Consequently, the balance between H3K27me3 and H3K4me3 is disrupted, leading to an overall shift in the epigenetic state toward gene suppression. Temporary inhibition of methionine cycle enzymes can lead to a sustained reduction in tumor potential, mainly due to changes in cellular methylation resulting from the depletion of SAM, a crucial substrate for methylation reactions (50, 52). Therefore, we can target SAM by reducing its production upstream and inhibiting its methyltransfer downstream. Once the active methyl groups are restricted, this abnormal methylation state is inevitably affected.

Methionine restriction can modulate anti-tumor immune function by influencing histone methylation modifications. Studies indicate that competitive consumption of methionine by tumor cells can lead to methionine metabolic deficiency in CD4^+^ T cells, which in turn suppresses AMPK expression by reducing histone H3K79me2 methylation levels (53, 54). The inhibition of AMPK further activates endoplasmic reticulum stress and the transcription factor XBP1s, ultimately leading to upregulation of PD-1 expression and impairing T cell immune function. Conversely, methionine supplementation or exogenous activation of AMPK can restore H3K79me2 and AMPK levels, reduce PD-1 expression, and thereby enhance the anti-tumor immune response of CD4^+^ T cells. This mechanism reveals that AMPK plays a key intermediary role between methionine metabolism and the epigenetic regulation of PD-1, and can be regarded as a metabolism-dependent immune checkpoint.

AGI-24512 and AG-270 are novel MAT2A inhibitors developed in recent years, which function by affecting PRMT5 activity and RNA splicing. Compared to PF-9366 and cycloleucine, their cellular potency is improved by 3–6 orders of magnitude (55). Research has demonstrated that the novel MAT2A inhibitor exhibits comprehensive efficacy, including targeted reduction of intracellular SAM, selective inhibition of proliferation and tumor growth in MTAP-deficient cancer cells *in vivo* (55). Furthermore, the significant findings from preclinical studies provide robust support for the initiation of related clinical trials.

Promoter hypermethylation is a vital epigenetic mechanism that significantly influences cancer onset, progression, diagnosis, and treatment by silencing essential gene expression. Dysregulated histone methylation acts as an assistant, playing a synergistic role. Targeting the SAM synthesis pathway is a key focus in anticancer research due to cancer cells’ reliance on SAM for maintaining their epigenetic state.

### 3.2 Methionine restriction potentiates oxidative stress by impairing tumor cell antioxidant defenses

A state of oxidative stress arises when ROS are produced and eliminated at unequal rates, causing harm to cellular components. In normal cells, moderate oxidative stress can function in cell signaling, while excessive oxidative stress contributes to disease development. However, when the “poison” in normal cells appears in cancer cells, oxidative stress takes on a completely opposite role. In the absence of external intervention, cunning cancer cells utilize oxidative stress as a signal to promote their own survival, proliferation, and spread (56). They activate multiple pathways promoting cell growth and division by maintaining oxidative stress at a controllable level, drive genetic instability and evolution, promote invasion and metastasis, and shape an immunosuppressive microenvironment (56–58). Conversely, excessive oxidative stress causes severe damage to cancer cells, forcing them to passively upregulate antioxidant defenses. If the excess ROS cannot be neutralized, it leads cancer cells to death. Therefore, they have developed a sophisticated system to regulate ROS levels, maintaining them within a “sweet spot” range beneficial to themselves: upregulating the antioxidant system, metabolic reprogramming, and activating adaptive responses (59). For example, in cancer cells, the antioxidant system is upregulated through the activation of transcription factors like NRF2, resulting in the increased production of antioxidants such as glutathione (GSH) and superoxide dismutase (SOD) to neutralize excess reactive oxygen species and prevent toxicity (60).

Homocysteine generated from methionine metabolism can enter the trans-sulfuration pathway, ultimately producing cysteine. The synthesis of GSH is limited by the availability of its rate-limiting substrate, cysteine. Consequently, the ability of this essential antioxidant to neutralize ROS is compromised when methionine is restricted (61). MR decreases homocysteine production, reducing cysteine and GSH supply and significantly lowering intracellular GSH levels. This can induce chronic inflammation and genomic instability, thus forming a pro-tumor microenvironment (62, 63). Cancer cells inherently produce excess ROS under rapid metabolism, making them more sensitive to GSH depletion. As a key member of the antioxidant system, GSH has been proven to be a potential target for cancer therapy (59, 64, 65). Therefore, methionine restriction indirectly elevates oxidative stress levels by weakening the cellular antioxidant defense system, which is particularly pronounced in certain highly metabolically active cancer cells. The key mechanism lies in the fact that reduced methionine concentration in the culture medium directly restricts glutathione synthesis, thereby impairing the cell’s ability to clear reactive oxygen species. For instance, in triple-negative breast cancer cells, methionine restriction rapidly and transiently increases intracellular reactive oxygen species levels, accompanied by a swift depletion of glutathione and a decline in the ratio of reduced to oxidized glutathione (66). These changes collectively indicate that methionine restriction does not directly “generate” oxidative stress; rather, by lowering the levels of glutathione-a core antioxidant molecule-it diminishes the cell’s capacity to counteract oxidative damage, thereby indirectly exacerbating the state of oxidative stress. This makes TNBC cells more reliant on the thioredoxin reductase (TXNRD) antioxidant pathway, creating a vulnerability to oxidative stress. The combined use of MR and the TXNRD inhibitor auranofin demonstrates notable antitumor effects in PDX TNBC mouse models (59). Research indicates that MR can trigger cancer cell death by modulating dysfunctional mitochondrial autophagy. A methionine-restricted diet can suppress tumor growth in cholangiocarcinoma cells, with enhanced inhibition observed upon overexpression of four mitochondrial protein-encoding genes: Succinate Dehydrogenase Complex Assembly Factor 2 (SDHAF2), Mitochondrial Ribosomal Protein S34 (MRPS34), Mitochondrial Ribosomal Protein L11 (MRPL11), and Cytochrome c Oxidase Subunit 8A (COX8A) (67). This indicates that methionine deprivation imposes further stress on mitochondrial function, thereby creating a synergistic cancer-suppressing effect with the functions of these genes. In gastric cancer cells, MR activates BNIP3 gene expression by downregulating lncRNA PVT1 expression and interacting with DNMT3, thereby activating mitophagy and inducing cell death (41).

In summary, methionine restriction selectively exacerbates the oxidative stress in cancer cells by targeting their antioxidant metabolic vulnerability-particularly through disrupting glutathione synthesis-thereby ultimately inducing cell death. This mechanism not only reveals a crucial pathway for tumor metabolic intervention but also provides a theoretical foundation for combination therapies targeting the redox system.

### 3.3 Methionine restriction exerts its antitumor effects by polyamine metabolism inhibition and metabolic interference

The high demand for methionine in cancer cells is sustained *in vivo* not only from dietary sources but also from limited methionine cycling and salvage pathways, namely remethylation and the MTA salvage pathway. Remethylation primarily refers to the process where the methionine intermediate metabolite homocysteine is reconverted into methionine. This process relies on sufficient vitamin B12 and folate, regenerating methionine through remethylation by methionine synthase (MS). Methylmalonic aciduria and homocystinuria (MMACHC) is a cytosolic chaperone for cobalamin (vitamin B12) and can be converted into the active cofactor methylcobalamin, which participates in methionine synthesis (68). MMACHC is essential for endogenous methionine synthesis as it directs vitamin B12 to methionine synthase (MS) in the cytoplasm. Melanoma MEWO cells are a special cell line that is not methionine-dependent, but when their MMACHC gene undergoes an epimutation, MEWO exhibits a methionine-dependent (MEWO-LC1) phenotype (69). An epimutation resulting from abnormal antisense transcription of the adjacent peroxiredoxin 1 (PRDX1) gene causes this condition. Alterations in the PRDX1 gene have been reported in individuals with CblC disease (methylmalonic aciduria and homocystinuria, cblC type)—a disorder that can be caused by compound heterozygous mutations or, notably, by an epimutation in the MMACHC promoter (70). Patients with the MEWO type might be candidates for MMACHC-targeted silencing, increasing the therapeutic targeting of MR. Furthermore, homocysteine can be converted back into methionine through the utilization of betaine, a metabolite of choline, which acts as a methyl donor in a reaction catalyzed by betaine homocysteine methyltransferase (BHMT). This pathway operates independently of folic acid and vitamin B12 and serves as a crucial supplementary route in the methionine cycle. The expression of BHMT is highly tissue-specific, predominantly concentrated in the liver, thereby designating the liver as the central “factory” for this pathway. This localization partially elucidates the strong link between methionine metabolism and liver cancer. Importantly, research has indicated that BHMT also plays a significant role in methionine recycling in extrahepatic tissues, such as in breast cancer. Choline serves as a direct precursor for the synthesis of phosphatidylcholine (PC) in the endoplasmic reticulum and can also be metabolized into betaine, facilitating the conversion of homocysteine (Hcy) to methionine and thereby sustaining the methionine cycle. Consequently, in conditions of methionine deficiency, the metabolic pathway of choline undergoes compensatory adjustments, resulting in a marked reduction in the levels of its derivatives, phosphatidylcholine (PC) and phosphatidylethanolamine (PE) (71–73).

**Figure 2:**
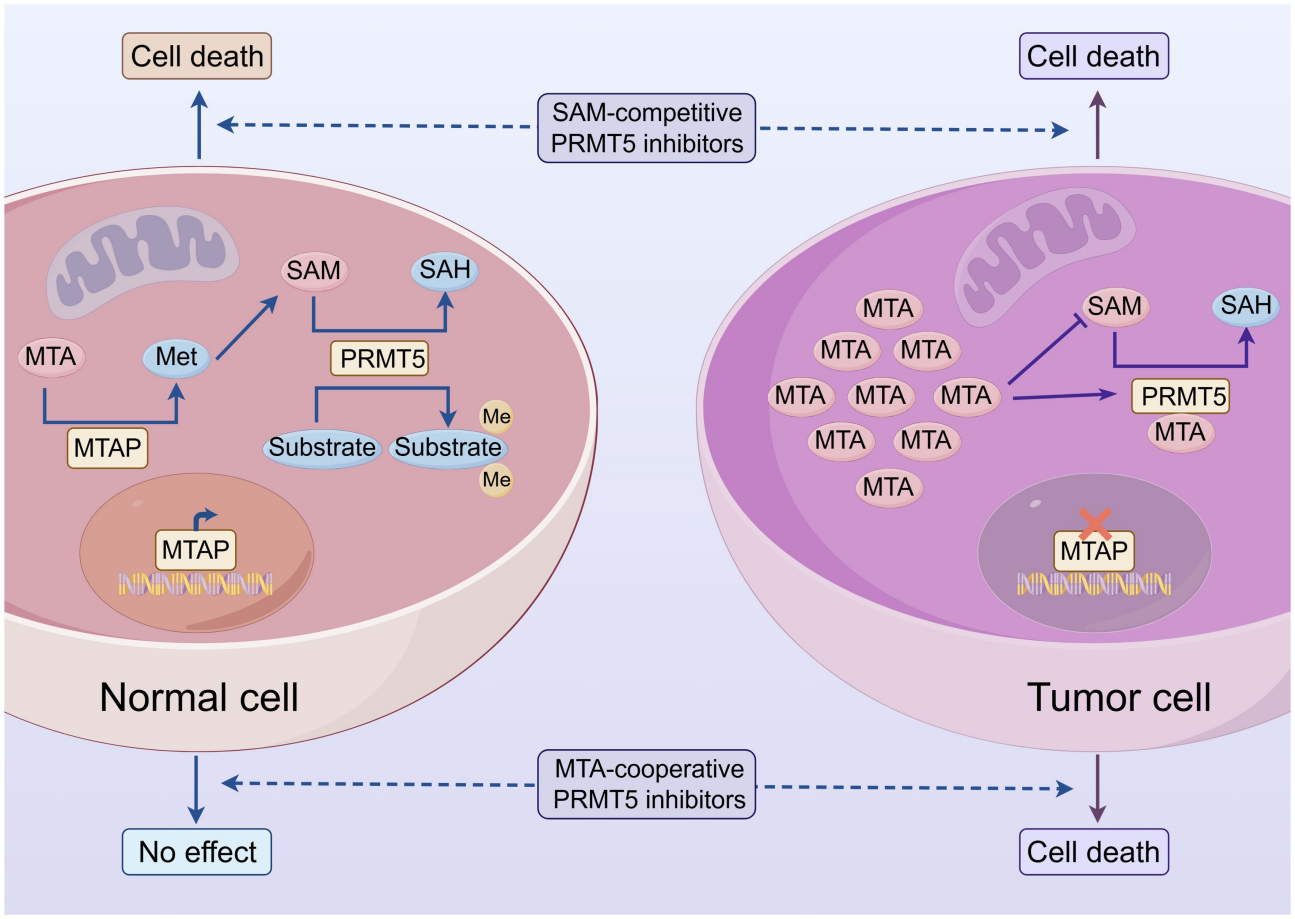
Tumors with MTAP deletion are synthetically lethal with PRMT5 inhibition. Methylthioadenosine (MTA), methionine (Met), S-adenosylmethionine (SAM), S-adenosylhomocysteine (SAH), methylthioadenosine phosphorylase (MTAP), protein arginine methyltransferase 5 (PRMT5), methylthioadenosine (MTA).

SAM functions as an essential precursor in the biosynthesis of polyamines, including spermine and spermidine (74). Polyamines are crucial for sustaining elevated protein synthesis, DNA replication, and cell proliferation rates, underpinning the rapid growth of cancer cells (75). Consequently, when the SAM production rate decreases, polyamines are inevitably affected. Approximately 15% of cancers (such as glioma, pancreatic cancer) have MTAP gene deletion, preventing them from salvaging MTA (76–78) (Figure 2). The polyamine metabolite MTA is an important precursor for the methionine salvage pathway. If the intake of exogenous methionine is reduced, the efficiency of the methionine cycle will significantly decline. Peter Kalev et al. The study primarily utilized HCT116 MTAP^−^/^−^ (MTAP-deficient) and HCT116 WT (wild-type) cells, as well as various tumor-derived and gene-knockout cell lines like HAP1 FANCI^−^, HAP1 FANCD2^−^, and HAP1 FANCL^−^, to examine the impact of MR on cancer cells (79). They used the MAT2A inhibitor AGI-24512 to simulate MR conditions, treating 316 different tumor-derived cell lines, and found that it inhibited the proliferation of most MTAP-deficient cell lines. AG-270 is a product structurally optimized based on AGI-24512, with high oral bioavailability and metabolic stability. It significantly lowers intracellular SAM levels, selectively inhibits the proliferation of MTAP^−^/^−^ cells, and decreases symmetric dimethylarginine (SDMA) marks. *In vivo* experiments showed that oral AG-270 significantly reduced tumor growth in MTAP^−^/^−^ xenografts, achieving a tumor growth inhibition (TGI) of 75% (*p* < 0.01), with no anti-proliferative effect on WT tumors; in various patient-derived xenograft (PDX) models, AG-270 reduced tumor growth in MTAP-deficient PDX models and had a selective impact on PRMT5 activity dependent on MTAP deletion. The above experimental results are consistent with the view that MTAP deletion and MAT2A inhibition have a synthetic lethal effect in cancer.

In summary, the metabolic characteristics of methionine in different types of tumors have been conducted and presented in Table 1.

**Table 1:** Metabolic characteristics of methionine in different types of tumors.

Tumor type	Key metabolic features	Therapeutically vulnerable pathways	Clinical implications	References
Gastric cancer	Long non-coding RNA PVT1 mediates promoter hypermethylation of BNIP3; impaired mitophagy.	PVT1-DNMT3 axis; methionine deprivation combined with HDAC2 inhibitors.	Activates BNIP3-dependent apoptosis; inhibits migration, invasion, and metastasis.	(41, 43)
Lung cancer	Coexistence of genome-wide hypomethylation and promoter hypermethylation of specific tumor suppressor genes (e.g., DAPK, RASSF1A); enhanced MAT2A-related metabolism.	Short-term methionine restriction (MR); ROS inducers; MAT2A inhibitors (e.g., PF-9366).	Targets cancer stem cells; reduces tumor malignancy; enhances chemosensitivity.	(31, 44–46)
Mixed-lineage leukemia (MLLr)	Significant overexpression of MAT2A; histone hypermethylation (e.g., H3K4me3, H3K79me2).	MAT2A-targeted therapy (PF-9366); combination with BET inhibitors.	Addresses treatment challenges in high-risk subtypes; induces differentiation.	(48–50)
Triple-negative breast cancer (TNBC)	Rapid and transient glutathione depletion; increased reliance on the thioredoxin reductase (TXNRD) pathway.	MR combined with TXNRD inhibitors (e.g., auranofin); NRF1/ATF2 inhibitors.	Generates 10-fold sensitivity in H-Ras-transformed cells; validated in PDX models.	(59, 66)
Glioma / pancreatic cancer	High frequency of MTAP co-deletion, leading to defective methionine salvage pathway.	MAT2A inhibitors (AG-270); MTA-cooperative PRMT5 inhibitors (AMG 193, MRTX1719).	Provides a synthetic lethal therapeutic strategy for MTAP-deleted tumors.	(5, 76, 98, 99)
Melanoma (MeWo Type)	Epigenetic silencing of the MMACHC gene, resulting in a methionine-dependent phenotype.	Targeted silencing of MMACHC or epigenetic modulators.	Converts non-dependent cells into methionine-dependent phenotype, expanding the target population for MR.	(69, 70)
Colorectal cancer (CRC)	Upregulation of the m^6^A reader YTHDF1, which promotes translation of immunosuppressive proteins (e.g., PD-L1).	Methionine restriction; genetic knockout or inhibition of YTHDF1.	Reverses CD8^+^ T cell exhaustion by blocking m^6^A-dependent translational reprogramming, enhancing anti-PD-1 efficacy.	(47)
Hepatocellular carcinoma / intrahepatic cholangiocarcinoma	MAT2A-dependent cancer stem cell maintenance; overexpression of specific mitochondrial genes (e.g., SDHAF2, MRPS34).	MAT2A-targeted therapy; chemosensitizers.	Overcomes high recurrence (5-year survival <20%); synergizes with mitochondrial gene overexpression to suppress tumors.	(36, 67)

BNIP3, BCL2/adenovirus E1B 19 kDa interacting protein 3; DNMT3, DNA methyltransferase 3; HDAC2, histone deacetylase 2; DAPK, death-associated protein kinase; RASSF1A, Ras association domain family member 1A; MAT2A, methionine adenosyltransferase 2A; ROS, reactive oxygen species; BET, bromodomain and extra-terminal; TXNRD, thioredoxin reductase; NRF1, nuclear respiratory factor 1; ATF2, activating transcription factor 2; MTAP, methylthioadenosine phosphorylase; PRMT5, protein arginine methyltransferase 5; PDX, patient-derived xenograft; MMACHC, methylmalonic aciduria and homocystinuria cblC type; m^6^A, N^6^-methyladenosine; YTHDF1, YTH N^6^-methyladenosine RNA binding protein F1; PD-L1, programmed death-ligand 1; SDHAF2, succinate dehydrogenase complex assembly factor 2; MRPS34, mitochondrial ribosomal protein S34.

## 4 The current landscape and future directions of methionine restriction for cancer treatment

### 4.1 Pharmacotherapy

Promoter hypermethylation, a key feature of tumorigenesis, primarily leads to the inactivation of tumor suppressor genes (80). For example, genes such as p16INK4a/CDKN2A, RASSF1A, and APC are silenced after promoter hypermethylation, leading to malignant cell proliferation and the acquisition of key characteristics of tumor cells (81, 82). SAM, produced by MAT, serves as the primary methyl donor in cells. It provides methyl groups via protein methyltransferases (such as PRMT5) to catalyze relevant intracellular reactions (DNA methylation, histone methylation), thereby regulating gene expression. It participates in polyamine synthesis, including compounds like spermine and spermidine. Polyamines are crucial for sustaining elevated protein synthesis, DNA replication, and cell proliferation, underpinning the rapid growth of cancer cells. Therefore, cancer cells urgently require sufficient methionine to synthesize SAM. Targeting the SAM synthesis pathway has become one of the most promising anti-cancer strategies. Next, we will discuss three drug types related to SAM targeting: MAT enzymes, MAT2A inhibitors, and PRMT5 inhibitors.

Soda et al. first utilized methionine *γ*-lyase, an enzyme, for cancer therapy in a mouse model in 1973 (83). And Tan et al. subsequently developed recombinant methioninase (rMETASE) (84). This enzyme was very effective at degrading methionine in tumor-bearing mouse models. In 2017, oral administration of o-rMETase demonstrated significant efficacy in patient-derived orthotopic xenograft (PDOX) models for multiple cancers, such as sarcoma and triple-negative breast cancer (85–90). To date, several late-stage cancer patients have been treated with o-rMETase as part of clinical research. Clinical investigations have demonstrated that oral administration of o-rmetase exhibits favorable safety and therapeutic potential in individuals with advanced cancer. In numerous cases involving ovarian, prostate, pancreatic, and rectal cancers, as well as high-grade glioma and metastatic breast cancer, the combination of o-rmetase with a low methionine diet or standard therapy has been shown to effectively reduce serum levels of tumor markers such as PSA, CEA, and CA19-9. Additionally, this treatment strategy has been associated with a reduction in tumor volume and the achievement of long-term disease stability, with no significant adverse reactions reported (91–96).

Back in the 1960s-70s, scientists discovered that cycloleucine could inhibit MAT activity, particularly MAT2A (97). Cycloleucine impedes S-adenosylmethionine (SAM) production by competing with methionine for the MAT2A enzyme binding site, thereby obstructing methionine’s interaction with ATP (97). However, because cycloleucine exhibited relatively broad inhibitory effects in cell and animal experiments, potentially leading to significant off-target effects and systemic toxicity, it is not suitable as an ideal clinical drug. Subsequently, MAT2A inhibitors such as PF-9366, AGI-24512, and AG-270 emerged. AG-270 is FDA-approved for clinical trials targeting advanced solid tumors or lymphomas with MTAP deletion (NCT03435250). Researcher Mrinal Gounder et al. The Phase I clinical trial of AG-270/S095033 for advanced malignant tumors was reported (79). A cohort of 40 patients diagnosed with advanced solid tumors or lymphomas was enrolled in the study, primarily selected based on the presence of a homozygous deletion of CDKN2A (35 patients) or reduced expression of MTAP (5 patients). Regarding efficacy, administration of ag-270/s095033 demonstrated a dose-dependent reduction in plasma SAM concentration by 65–70% within the dosage range of 50–200 mg. However, efficacy diminished to 54% at a dosage of 400 mg once daily (QD). Pharmacokinetic analysis indicated that a 200 mg QD dosage was well-tolerated, with exposure levels increasing proportionally within the 50–200 mg range, although a saturation trend was observed at 400 mg QD. Among the 40 patients, 2 experienced partial responses and 5 had stable disease for at least 16 weeks, achieving a disease control rate of 17.5%. This indicates that AG-270/S095033 has certain anti-tumor activity, providing pharmacokinetic and pharmacodynamic evidence for MAT2A inhibition. This is the first report of Phase I results for AG-270/S095033 monotherapy in cancer, providing human data for MAT2A inhibitors in treating MTAP-deleted cancers. The MAT2A inhibitor AG-270/S095033 decreases S-adenosylmethionine (SAM) levels and suppresses protein arginine methyltransferase 5 (PRMT5) activity in tumors lacking MTAP. This results in decreased symmetric dimethylarginine (SDMA) residues on proteins associated with mRNA splicing, leading to cell death. However, the trial results were not entirely satisfactory, which we believe is due to insufficiently strict patient screening. Using CDKN2A deletion as a proxy for MTAP deletion in the study was inaccurate, leading to the enrollment of many patients insensitive to AG-270/S095033, a finding consistent with AG-270 cell experiments.

In summary, MAT2A inhibitors exert their selective antitumor effect in MTAP-deleted cancers through a defined mechanism: MAT2A inhibition causes MTA accumulation, which acts as a natural competitor to SAM for PRMT5 binding, resulting in global hypomethylation and disrupted proliferation. Consequently, MTAP-intact tumors remain unresponsive. This logically raises a pivotal question: can PRMT5 inhibitors effectively address this therapeutic gap in non-MTAP-deleted tumors?

PRMT5 is recognized as a therapeutic target in tumors characterized by MTAP deletion (98). The PRMT5 inhibitor AMG 193 has demonstrated promising efficacy in clinical trials. In 2024, Rodon J et al. published findings from a Phase I dose-finding study of AMG 193, involving 80 patients who received doses ranging from 40 to 1,600 mg once daily (o.d.) or 600 mg twice daily (b.i.d.) (99). The research determined that the highest dose tolerated is 1,200 mg per day. Across the patient cohort, AMG 193 exposure was consistent, with 10% of participants experiencing dose-limiting toxicities, including vomiting, fatigue, hypersensitivity, and hypokalemia, at doses of 240 mg or higher. Among the 42 participants assessable for efficacy at active and tolerated doses, 21.4% showed an objective response, with a 95% confidence interval ranging from 10.3 to 36.8%. Antitumor activity was observed across eight solid tumor types. Complete inhibition of PRMT5 within tumors and molecular responses, like the elimination of circulating tumor DNA, were confirmed at doses of 480 mg or more. AMG 193 was generally well tolerated, with no clinically significant myelosuppression reported.

### 4.2 Dietary intervention

Diet has a long-term effect on health, and dietary changes are often employed to address diseases with a metabolic origin (17, 100). Reducing methionine in the diet, a vital amino acid for one-carbon metabolism, has proven to extend lifespan and boost metabolic health (101). It is certain that pharmaceutical intervention in the metabolic process of methionine in the body can produce controllable therapeutic effects on MTAP-deficient tumor cells (102). Due to their uncontrolled proliferation and epigenetic reprogramming, tumor cells develop an “addictive” and massive demand for the methyl donor SAM. Simultaneously, their own methionine salvage mechanisms are often defective (e.g., MTAP deletion), preventing them from being “self-reliant” and forcing them to highly depend on exogenous methionine intake to maintain their malignant characteristics. Therefore, dietary intervention is a potential modality for adjuvant therapy in cancer patients, and it is safer compared to the potential off-target effects of pharmaceutical interventions (such as AG-270) (103).

Diet is closely related to human health. Currently, there are two main dietary patterns: plant-based diets and animal-based diets, which are based on foods derived from plants and animals, respectively. A 2021 study reported that a higher plant-based diet index was highly statistically associated with lower all-cause mortality in patients with colorectal cancer (104). Furthermore, prior to this, a dietary intervention study conducted among breast cancer survivors (increasing the intake of vegetables, fruits, and grains, and controlling fat intake to less than 20% of total caloric intake) also demonstrated that a diet high in plant-based foods was significantly associated with reduced mortality (105). Plant-based foods and animal-based foods differ considerably in the content of certain amino acids, such as methionine. Methionine content is relatively low in most plants, with the exception of some grains and legumes. Therefore, a plant-based diet results in a relatively lower intake of methionine, which represents a healthier dietary approach for cancer patients.

The study by Gao et al. published in Nature involved 6 healthy participants on a low-methionine diet for 3 weeks, achieving a 60% reduction in serum methionine levels, modifying associated metabolite profiles, and showing good tolerability (17). This was the first demonstration in humans of the feasibility and reproducible metabolic effects of a methionine-restricted diet. Subsequently, more researchers conducted several small phase I/II clinical trials targeting advanced solid tumors (106–108). When combined with chemotherapy (e.g., 5-FU, cisplatin, temozolomide) or radiotherapy, the low-methionine diet generally demonstrated manageable safety without unexpected severe toxic side effects (107–109). Consistent with animal experiments, patients’ plasma methionine and related metabolite (e.g., SAM) levels decreased significantly (106). In some patients insensitive or resistant to conventional chemotherapy, cases of stable disease (SD) or even partial response (PR) were observed after combining MR. For instance, in patients with colorectal cancer and glioblastoma, MR diet combined with chemotherapy showed better disease control rates. Studies suggest that MR depletes SAM, inhibiting the activity of DNA repair proteins (such as MGMT), thereby sensitizing cancer cells to DNA-damaging chemotherapy and radiotherapy (110).

### 4.3 The potential of combination therapy

Due to the complex pathogenesis of tumors, combination therapy is undoubtedly the future direction of cancer treatment. Currently, clinical methionine restriction is achieved through dietary, enzymatic, and pharmaceutical means, often combined with sensitizing approaches like chemotherapy and radiotherapy for patient treatment. It has been confirmed to be non-toxic, but may cause some adverse reactions like nausea and vomiting. Patients with MTAP deletion tend to have relatively better treatment outcomes. Combined with diagnostic methods for precise screening of MTAP status, the future holds promise for the synergistic application of personalized dietary intervention and targeted therapy, offering a more specific and lower-toxicity comprehensive treatment plan for metabolically vulnerable tumors.

Antitumor immunotherapy has been one of the breakthroughs in the field of cancer in recent years. Its core idea is no longer to directly attack cancer cells, but to lift the suppression of cancer cells on the immune system and enhance the patient’s own immune system to recognize and eliminate tumor cells. Both T cells and tumor cells can express the Solute Carrier Family 43 Member 2 (SLC43A2), a key methionine transporter; however, due to the methionine addiction of tumor cells, they highly express the SLC43A2 transporter. Consequently, tumor cells can engage in fierce competition with immune cells, acquiring large amounts of methionine (111–113). In cancer cells, P53 can promote the uptake and utilization of methionine by cancer cells, intensifying their competition with T cells (114). Methionine deficiency in T cells leads to their functional exhaustion, resulting in consequences such as immune escape that affect cancer progression. Due to their typically heightened methionine addiction and frequent formation of an immunosuppressive microenvironment, methionine restriction (MR) may primarily exert its effect by directly inhibiting tumor growth in p53-deficient tumors. In contrast, for tumors carrying wild-type p53, where the microenvironment may rely more heavily on functional T cells, MR requires careful evaluation to avoid impairing antitumor immunity. Research indicates that deletion of 9p21 can lead to an immunologically “cold” tumor phenotype, characterized by reduced tumor-infiltrating leukocytes, impaired recruitment and activation of immune cells, decreased PD-L1 expression, and activation of immunosuppressive signaling (115). Patients with such tumors exhibit significantly lower response rates to immune checkpoint therapy and poorer prognosis. Therefore, converting “cold” tumors into “hot” tumors represents a crucial therapeutic strategy. In osteosarcoma, MTAP deficiency contributes to a “cold” tumor microenvironment marked by insufficient CD8^+^T cell infiltration, thereby hindering the effective initiation of antitumor immune responses (116). However, methionine restriction or MAT2A inhibitors (e.g., SCR6639) can upregulate PD-L1 expression by activating the transcription factor IKZF1, promote CD8^+^T cell infiltration, and consequently enhance the efficacy of immune checkpoint therapy. In essence, methionine restriction helps overcome the immunosuppressive state by increasing T cell infiltration, thereby effectively mobilizing and enabling antitumor immunity to exert its function.

Besides enhancing the sensitivity to traditional radiotherapy and chemotherapy, MR can also boost antitumor immune responses by modulating T cell activity in the tumor microenvironment (117). Currently, the most widely and successfully applied immunotherapies in clinical practice primarily include immune checkpoint inhibitors and Chimeric Antigen Receptor (CAR) T-cell therapy. The high expression of PD-L1 on the surface of tumor cells can inhibit the attack capability of T cells; therefore, PD-L1 inhibitors can be used for immunotherapy in relevant cancer patients. Recent studies have shown that in patients with triple-negative breast cancer, PD-L1 inhibitors can prolong survival both in the intention-to-treat population and in the PD-L1-positive subgroup (118, 119). Studies have demonstrated that MR can reduce the expression of the PD-L1 gene in cancer cells, and the therapeutic effect of clinically combining MR with inhibitors targeting PD-L1 and cytotoxic T-lymphocyte-associated protein 4 (CTLA-4) is significantly greater than that of single-agent therapy (120). However, while methionine restriction (MR) can inhibit tumor growth, it may also exert negative effects on anti-tumor immunity, which is particularly notable in CAR-T therapy. Studies have shown that MR-induced reduction in S-adenosylmethionine (SAM) affects the activity of m5C methyltransferases, leading to downregulation of m5C RNA modification (115). This, in turn, reduces the memory phenotype of T cells and decreases the expression of key effector molecules such as Natural Killer Cell Granule Protein 7 (NKG7), ultimately impairing the cytotoxicity of CAR-T cells (121). It is noteworthy that the immunoregulatory role of methionine metabolism is highly context-dependent. Methionine/SAM metabolism can promote cGAS methylation via the SUV39H1-UHRF1 axis, thereby actively suppressing the cGAS-STING pathway-a mechanism contributing to tumor immune evasion (122). Correspondingly, studies indicate that short-term methionine restriction can lower cGAS methylation levels, thereby relieving this suppression and reactivating the cGAS-STING pathway to exert anti-tumor immune effects; however, prolonged methionine deprivation or loss of methylation may lead to complex outcomes (122, 123).

Furthermore, a recent *in vivo* study revealed another pathway through which methionine restriction (MR) impairs immunity: by inducing systemic sulfur deficiency and reducing hydrogen sulfide production by the gut microbiota, thereby suppressing endogenous T cell-mediated anti-tumor immunity. This resulted in accelerated tumor progression and acquired resistance to immunotherapy in immunocompetent mice (117). Of note, the same study proposed a potential countermeasure-supplementation with hydrogen sulfide donors or cysteine in mouse models effectively alleviated MR-induced immunosuppression. This finding opens a new direction for designing more precise dietary intervention strategies in cancer.

In the future, the combination of MR and anti-tumor immunotherapy may achieve a “1 + 1 > 2” effect. Although challenges remain, with the advancement of precision medicine concepts and the development of novel drugs, MR is highly likely to become an important “precision weapon” in the comprehensive cancer treatment toolbox in the coming years, particularly offering new hope for patients carrying specific metabolic defects (such as MTAP deletion).

The current clinical Intervention strategies targeting methionine metabolism are summarized in Table 2.

**Table 2:** Comparison of clinical intervention strategies targeting methionine metabolism.

Intervention strategy	Representative drug/method	Mechanism of action	Development stage	Advantages	Challenges	References
Dietary intervention	Low-methionine diet	Directly restricts exogenous methionine, reducing SAM. Its effects are mediated through a complex downstream network: Global Epigenetic Reprogramming: SAM depletion differentially affects various methyltransferases; for example, it preferentially inhibits MLL1 (H3K4me3) while relatively maintaining EZH2 (H3K27me3) activity, driving gene silencing programs. Impact on Multiple Methyltransferases: In addition to PRMT5, it also inhibits PRMT1 (affecting RNA processing, etc.) and other SAM-dependent enzymes. Oxidative Stress & Immune Modulation: As previously described.	Clinical Phase I/II, combined with chemo/radiotherapy; applicable to various solid tumors.	High safety profile, potentially low cost.	Poor patient compliance, difficult to precisely control and maintain.	(17, 51, 53, 54, 106–108)
Enzymatic degradation	Oral recombinant methioninase (o-rMETase)	Degrades methionine in the gastrointestinal tract, systemically reduces circulating methionine levels, acting similarly to but more controllably than dietary restriction.	Clinical case reports, combined with chemo/radiotherapy.	Oral convenience, can supplement dietary restriction.	Requires larger clinical trials to verify efficacy.	(85–96)
MAT2A inhibitor	AG-270, PF-9366	Inhibits SAM synthesis. In MTAP-deficient tumors, it primarily inhibits PRMT5 via MTA accumulation. Concurrently, the global reduction in SAM also affects: The balance of histone methyltransferases (EZH2 vs. MLL1). The activity of other PRMT family members (e.g., PRMT1). This represents the combined outcome leading to synthetic lethality and epigenetic effects.	AG-270 in Phase I trials for MTAP-deleted advanced solid tumors/lymphoma.	High selectivity for MTAP-deficient tumors, good oral bioavailability.	Primarily effective against MTAP-deleted tumors; potential resistance mechanisms exist.	(51, 55, 79, 103)
PRMT5 inhibitor	AMG 193	Directly inhibits PRMT5 methyltransferase activity, affecting key cellular functions such as mRNA splicing. Synergizes with endogenous MTA in MTAP-deleted tumors for enhanced efficacy.	Clinical Phase I/II for various MTAP-deleted solid tumors.	High efficacy, objective responses observed in various MTAP-deleted cancers.	Potential drug resistance, requires precise biomarkers.	(98, 99)
Combined immunotherapy strategy	MR + Immune Checkpoint Inhibitors	Metabolic-Immune Co-regulation: Reduces tumor SAM levels via MR, thereby downregulating immunosuppressive protein synthesis, reversing T cell exhaustion, and activating the cGAS-STING pathway. This converts “cold” tumors into “hot” ones, synergizing with anti-PD-1/PD-L1 therapy.	Preclinical and early clinical exploration phase	Synergistically overcomes monotherapy resistance and extends clinical benefit.	Immune effects are dose- and timing-sensitive, lacking standardized combination guidelines.	(47, 53, 117, 120, 122)

SAM, S-adenosylmethionine; o-rMETase, oral recombinant methioninase; MAT2A, methionine adenosyltransferase 2A; MTA, methylthioadenosine; MTAP, methylthioadenosine phosphorylase; EZH2, Enhancer of Zeste Homolog 2; MLL1, Mixed-Lineage Leukemia 1; PRMT1, Protein Arginine Methyltransferase 1; PRMT5, Protein Arginine Methyltransferase 5; AG-270, MAT2A inhibitor AG-270; PF-9366, MAT2A inhibitor PF-9366; PRMT5, protein arginine methyltransferase 5; AMG 193, PRMT5 inhibitor AMG 193; cGAS-STING, cyclic GMP-AMP synthase-stimulator of interferon genes; PD-1, programmed cell death protein 1; PD-L1, programmed death-ligand 1.

## 5 Conclusion

It should be noted that cancer cells’ dependency on methionine is not uniform. The extent of this dependency may be influenced by factors such as tumor type, genetic background (e.g., MTAP status), and the microenvironment. This review systematically demonstrates the potential of MR as an anticancer strategy: substantial evidence indicates that MR can effectively inhibit tumor growth. It can be achieved through dietary modulation, recombinant methioninase, or targeting pathways such as SAM synthesis, and has demonstrated significant efficacy with manageable safety in both preclinical and clinical studies, thus holding broad prospects in cancer therapy.

Building on this potential, to advance the clinical translation of MR, we propose the following key directions: First, achieving precise patient selection-given the heightened sensitivity of MTAP-deficient cancers to MR, MTAP status should be evaluated as a predictive biomarker to prioritize potential beneficiaries. Second, developing synergistic therapeutic strategies-inhibiting the SLC43A2 transporter can diminish the methionine advantage of tumor cells, thereby restoring the anti-tumor function of T cells; this strategy is mechanistically synergistic with immune checkpoint inhibitors (such as anti-PD-1/PD-L1 therapy), and the deep integration of the two holds promise for pioneering a new generation of treatment paradigms. However, the impact of MR on the immune system is context-dependent. Some studies suggest that MR may impair T-cell function under certain conditions, and long-term restriction could lead to complex metabolic feedback. Therefore, future research must, while promoting translation, deeply dissect the dual roles of MR in different immune microenvironments to mitigate potential risks.

Currently, MR has entered clinical exploration through tailored diets or pharmacological interventions. Although long-term strict dietary adherence poses challenges, optimized MR regimens remain a viable therapeutic option. Looking ahead, the core of advancing this field lies in “precision”: it is necessary to develop biomarker-based individualized treatment plans according to different cancer types and their specific metabolic contexts, thereby transitioning methionine-targeted therapy from concept to clinic and truly integrating it as a vital component of the precision oncology framework.
